# The H3K9 methyltransferase G9a is a marker of aggressive ovarian cancer that promotes peritoneal metastasis

**DOI:** 10.1186/1476-4598-13-189

**Published:** 2014-08-12

**Authors:** Kuo-Tai Hua, Ming-Yang Wang, Min-Wei Chen, Lin-Hung Wei, Chi-Kuan Chen, Ching-Huai Ko, Yung-Ming Jeng, Pi-Lin Sung, Yi-Hua Jan, Michael Hsiao, Min-Liang Kuo, Men-Luh Yen

**Affiliations:** Graduate Institute of Toxicology, National Taiwan University College of Medicine, Taipei, Taiwan; Departmant of Surgery, National Taiwan University Hospital, Taipei, Taiwan; Department of Oncology, National Taiwan University Hospital, Taipei, Taiwan; Cell Engineering Lab, Biomedical Technology and Device Research Labs, Industrial Technology Research Institute, Hsinchu, Taiwan; Department of Pathology, National Taiwan University Hospital, Taipei, Taiwan; Department of Obstetrics and Gynecology, Taipei Veterans General Hospital and National Yang-Ming University, Taipei, Taiwan; Institute of Clinical Medicine, National Yang-Ming University, Taipei, Taiwan; Genomics Research Center, Academia Sinica, Nankang, Taipei Taiwan; Department of Primary Care Medicine, National Taiwan University College of Medicine, Taipei, Taiwan; Department of Obstetrics/Gynecology, National Taiwan University College of Medicine, Taipei, Taiwan

**Keywords:** Histone methyltransferase, G9a, Ovarian cancer, Peritoneal metastasis

## Abstract

**Background:**

Ovarian cancer (OCa) peritoneal metastasis is the leading cause of cancer–related deaths in women with limited therapeutic options available for treating it and poor prognosis, as the underlying mechanism is not fully understood.

**Method:**

The clinicopathological correlation of G9a expression was assessed in tumor specimens of ovarian cancer patients. Knockdown or overexpression of G9a in ovarian cancer cell lines was analysed with regard to its effect on adhesion, migration, invasion and anoikis-resistance. In vivo biological functions of G9a were tested by i.p. xenograft ovarian cancer models. Microarray and quantitative RT-PCR were used to analyze G9a-regulated downstream target genes.

**Results:**

We found that the expression of histone methyltransferase G9a was highly correlated with late stage, high grade, and serous-type OCa. Higher G9a expression predicted a shorter survival in ovarian cancer patients. Furthermore, G9a expression was higher in metastatic lesions compared with their corresponding ovarian primary tumors. Knockdown of G9a expression suppressed prometastatic cellular activities including adhesion, migration, invasion and anoikis-resistance of ovarian cancer cell lines, while G9a over-expression promoted these cellular properties. G9a depletion significantly attenuated the development of ascites and tumor nodules in a peritoneal dissemination model. Importantly, microarray and quantitative RT-PCR analysis revealed that G9a regulates a cohort of tumor suppressor genes including *CDH1*, *DUSP5*, *SPRY4*, and *PPP1R15A* in ovarian cancer. Expression of these genes was also inversely correlated with G9a expression in OCa specimens.

**Conclusion:**

We propose that G9a contributes to multiple steps of ovarian cancer metastasis and represents a novel target to combat this deadly disease.

**Electronic supplementary material:**

The online version of this article (doi:10.1186/1476-4598-13-189) contains supplementary material, which is available to authorized users.

## Introduction

Epithelial ovarian cancer (OCa) has the highest mortality rate of all the gynecologic tumors [1]. Without effective screening, most OCa patients are diagnosed at an advanced stage with metastatic disease [2]. Furthermore, the metastatic pattern of OCa differs from the patterns of most other malignant epithelial diseases. Approximately 70% of OCa patients present with disease that has peritoneal metastases, in which tumor cells have spread beyond the ovaries [3]. Peritoneal metastases contribute substantially to morbidity because they have the capacity to affect multiple vital organs within the abdomen [4]. Unfortunately, very little is known about the mechanisms behind this process. The understanding of the molecular mechanisms that regulate the motility and invasive behavior of OCa cells is critical for improving the outcomes of patients with this devastating disease.

Epigenetic dysfunction plays a central role in the pathology of OCa [5]. Atypical modification of histones and dysregulated expression of histone-modifying enzymes have been found in OCa [6, 7]. G9a, a histone methyltransferase for lysine 9 of histone 3 (H3K9), was originally identified as a key histone methyltransferase (HMT) that mediates euchromatin gene silencing and is essential for early embryogenesis through regulating developmental gene expression [8, 9]. G9a has since been found to cooperate with transcription factors to regulate gene expression [10–12], and G9a-dependent H3K9 methylations have been shown to mediate epigenetic silencing of several tumor suppressor genes including *DSC3*, *MASPIN*, and *CDH1* [13, 14]. Subsequent studies have reported epigenetic activation of the serine-glycine biosynthetic pathway by G9a upon serine depletion in cancer cells [12] and have shown that G9a and H3K9 methylations were required to sustain cancer cell behaviors like hypoxia response, cell proliferation, metabolism, autophagy, cancer stemness, and epithelial-mesenchymal transition [12, 14–17]. Small-molecule inhibitors of G9a have also developed and their tumor suppressing effects have been observed [18, 19]. Recent studies have found G9a to be more highly expressed in various types of tumor tissue, including OCa, than in their non-cancerous counterparts [20], and we observed a significant correlation of G9a expression and patient survival in lung cancer in a previous study [21, 22]. Given that epigenetic regulations are capable of simultaneously controlling expression of a host of gene cohorts, G9a is predicted to regulate a cluster of genes affecting cancer behavior.

Although there exists an increasing body of evidence supporting the involvement of G9a in tumor development, the role of G9a in OCa remains obscure. In this study, we investigated the role of G9a in OCa progression and identified a G9a-regulated gene cohort. Our results indicate that G9a was highly correlated with metastatic properties of OCa and may promote OCa metastasis through simultaneous regulation of metastasis-related genes.

## Results

### Expression of G9a in tumors correlates with tumor progression and poor prognosis in OCa

**Figure 1:**
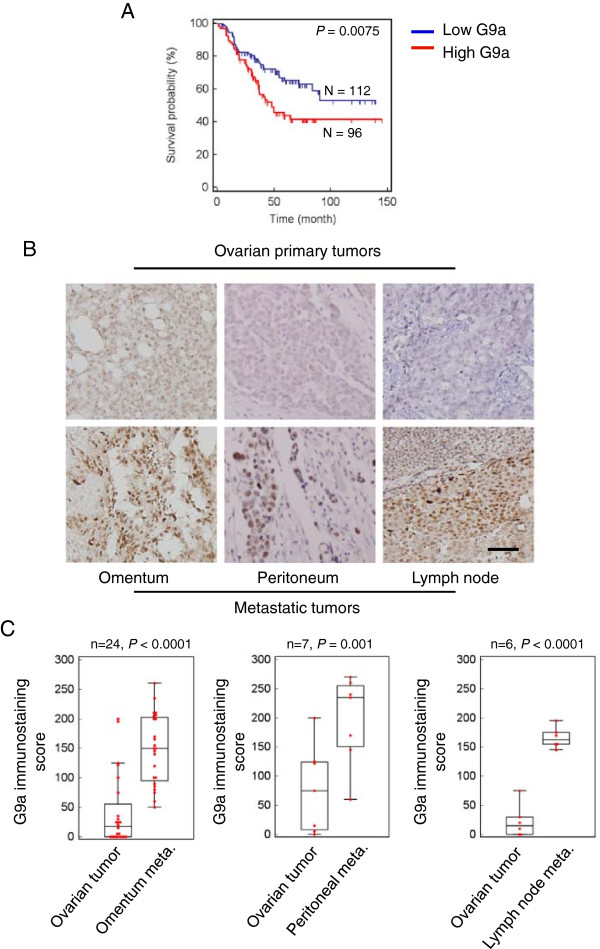
**G9a overexpression in omental metastatic lesions compared with the corresponding primary tumors. A**. Kaplan–Meier plot of the overall survival of 208 OCa patients, stratified by G9a expression. Low G9a: score of 0 or 1; high G9a: score of 2 or 3. Censored data marked in the plot as short vertical lines. **B**. Representative microphotographs of immunohistochemical staining of G9a in matched specimens of primary ovarian tumors and metastases from omentum, peritoneum, and lymph node. Scale bar: 100 μm. **C**. Plots depicting scores according to the immunohistochemical expression of G9a in primary ovarian tumors and metastases. 24 pairs of omental metastases, 7 pairs of peritoneal metastases, and 6 pairs of lymph node metastases were included in the analysis. The scores were calculated by intensity × percentage of stained cells. The association between G9a expression level and metastasis was analyzed using a paired *t*-test and displayed by box-plot.

We first examined G9a protein levels in 208 ovarian tumors by immunohistochemistry (IHC). Of the OCa specimens tested for G9a expression, 71.6% showed positive staining. The positive cases were divided into three score classes according to their staining intensity: low in 53 cases (25.5%), moderate in 48 cases (23%), and high in 48 cases (23%). G9a expression significantly correlated with late stage, high grade, and serous-type OCa (Additional file 1: Table S1). Furthermore, univariate analysis revealed that the presence of G9a expression, the histological grade, the histological subtype and the stage of the disease were all significantly associated with overall survival (Hazard ratio = 1.80, 1.72, 1.71, 4.72; *P* = 0.009, 0.01, 0.016 and <0.0001, respectively). Backward stepwise multivariate analysis revealed that disease stage was the only independent risk factor associated with overall survival (Hazard ratio = 2.57, *P* = 0.009, Table 1). The failure of G9a to be identified as an independent prognostic factor results from the significant correlation between G9a expression and stage of disease (correlation rho = 0.23, *P* = 0.0012). The Kaplan–Meier curve is presented in Figure 1A. The median survival interval of patients with high G9a expression was 48 months, and were not reached in patients with low G9a expression (*P* = 0.0075, log rank test). The correlation rho of the severity of G9a expression and the overall survival interval was -0.15 (*P* = 0.036, Spearman rank correlation test). These results suggest that G9a is a prognostic marker for OCa progression.

**Table 1:** **Univariate and multivariate analysis of potential prognostic variables**

Parameters	Comparison	Univariate analysis		Multivariate analysis	
		HR (95% CI)	***P***-value	HR (95% CI)	***P***-value
G9a score	Low (0, 1); High (2, 3)	1.80 (1.16 to 2.77)	0.009*	1.42 (0.84 to 2.40)	0.196
FIGO stage	pT1-pT2; pT3-pT4	4.72 (2.73 to 8.15)	<0.0001*	2.57 (1.27 to 5.22)	0.009*
Histological grade	G1; G2; G3	1.72 (1.14 to 2.60)	0.010*	1.32 (0.85 to 2.04)	0.214
Histological subtype	Serous; Non-serous	1.71 (1.11 to 2.63)	0.016*	0.89 (0.48 to 1.66)	0.729

NOTE: Cox proportional hazards regression was used to perform uni- and multivariant analysis for potentially important variates.

*Abbreviations*: *HR* hazard ratio, *CI*, confidence interval.

*Two-sided Cox proportional hazards regression using normal approximation.

### G9a expression is elevated in metastatic OCa tissue

The staging of OCa was primarily defined according to metastatic progression. To further evaluate the role of G9a during the progression of OCa, G9a expression was assessed by IHC analysis in individually matched samples from primary ovarian tumors and their corresponding metastatic tumors. Representative IHC photos of G9a staining are shown in Figure 1B. Relative to the matched primary tumor tissues, G9a expression was significantly higher in 24 sets of omental metastatic tumor tissue (Figure 1C, left panel). Furthermore, G9a expression was also significantly higher in peritoneal metastases (7 pairs, *P* = 0.001) and lymph node metastases (6 pairs, *P* < 0.0001) (Figure 1C, middle and right panels). Collectively, these results suggest that G9a expression increases during metastasis of OCa.

### G9a expression correlates with peritoneal metastasis-relevant traits in vitro

**Figure 2:**
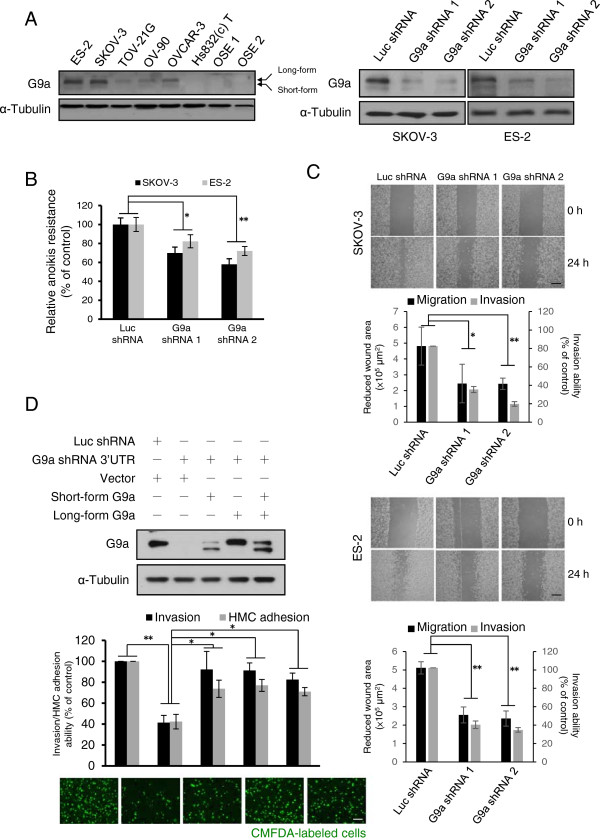
**G9a expression contributes to OCa cell adhesion, cell motility and anoikis resistance. A**. Left panel: protein expression of G9a in OCa cell lines (ES-2, SKOV-3, TOV-21G, OV-90 and OVCAR-3), cyst cell line Hs832(c)T and two OSE primary cells. Right panel: Knockdown efficiency of two G9a specific shRNAs in SKOV-3 and ES-2 cells. **B**. Anoikis assays of SKOV-3 and ES-2 cells infected with specific shRNAs as indicated. **C**. Migration and invasion ability of G9a knock down cells. Wound-healing assays and transwell invasion assays were used to determine the cell migration and invasion ability of two OCa cell lines, SKOV-3 and ES-2. Reduced wound area after 24 h was analyzed by ImageJ analysis software. Cells that invaded into the opposite site of transwell were counted and presented as% of control. Representative micrographs of wound-healing assays shown above the plot. Scale bar: 500 μm. **D**. SKOV-3 cells with G9a depletion by shRNA that target 3’UTR region of G9a mRNA were transfected with long-form and/or short-form G9a and subjected to transwell invasion and HMC adhesion assays after fluorescence labeled with CellTracker™ Green CMFDA. Scale bar: 100 μm. Results indicated are mean values of three independently repeated experiments done in triplicate and are displayed as mean ± SD. *P < 0.05, **P < 0.01, indicate a statistically significance by one way ANOVA followed by Bonferroni test.

In light of the observed correlation between tumor stage and G9a expression in clinical specimens, we examined the potential role of G9a in regulating metastasis-relevant traits. G9a was highly expressed in OCa cell lines (ES-2, SKOV-3, TOV-21G, OV-90 and OVCAR-3) compared with the benign ovarian cyst cell line, Hs832(c)T, and with primary ovarian epithelial cells (Figure 2A, left panel). G9a expression was especially high in two highly invasive cell lines, SKOV-3 and ES-2, compared with other tested cells. We next depleted G9a expression in SKOV-3 and ES-2 cells by lentiviral introduction of two specific shRNAs (Figure 2A, right panel). Cell proliferation was not significantly affected by G9a shRNAs within 72 h (Additional file 1: Figure S1). However, when these cells were placed in a detached environment, G9a knockdown cells showed increased cell death compared with control cells (Figure 2B), suggesting that the presence of G9a counteracts anoikis. Furthermore, G9a-depleted cells also grew smaller colonies in soft agar compared to the control cells (Additional file 1: Figure S2). Adhesion assays using plates coated with different extracellular matrix components were performed to evaluate the effect of G9a on the adhesion behavior of OCa cells. Although SKOV-3 and ES-2 cells exhibited different levels of adhesion against collagen I, fibronectin and Matrigel, adhesion was always significantly decreased when G9a expression was suppressed (Additional file 1: Figure S3). In contrast, G9a knockdown cells had the same ability to adhere to non-coated surfaces as the control cells, which suggests that the affected adhesion behavior was specific to the extracellular matrix components. Cell adhesion assay using the monolayer of human primary mesothelial cells further confirmed the requirement of G9a expression to sustain adhesion levels in SKOV-3 cells (Additional file 1: Figure S3).

As peritoneal implants require the invasion of previously adhered tumor cells into the peritoneum, cell migration/invasion capacity was then evaluated by wounding/invasion assays. G9a knockdown cells were less proficient than the control cells at closing an artificial wound created over a confluent monolayer (Figure 2C, black column). Transwell invasion assays also demonstrated that G9a depleted cells were significantly less invasive than control cells (Figure 2C, grey column). To further confirm the dependence of G9a expression on cell invasion and adhesion, we performed rescue experiments with two G9a isoforms of similar enzymatic activity, 165 kDa G9a-L and/or 140 kDa G9a-S [23]. Expression of both G9a isoforms brought the invasion and adhesion levels of G9a-depleted SKOV-3 cells back to normal (Figure 2D). Over-expression of G9a isoforms also slightly increased cell mobility and adhesion in low G9a-expressing OV-90 cells (Additional file 1: Figure S4). Taken together, these in vitro assays demonstrate that G9a expression is essential for maintaining the pro-peritoneal metastasis traits of G9a expressing OCa cells.

### G9a suppression inhibits OCa metastasis in vivo

**Figure 3:**
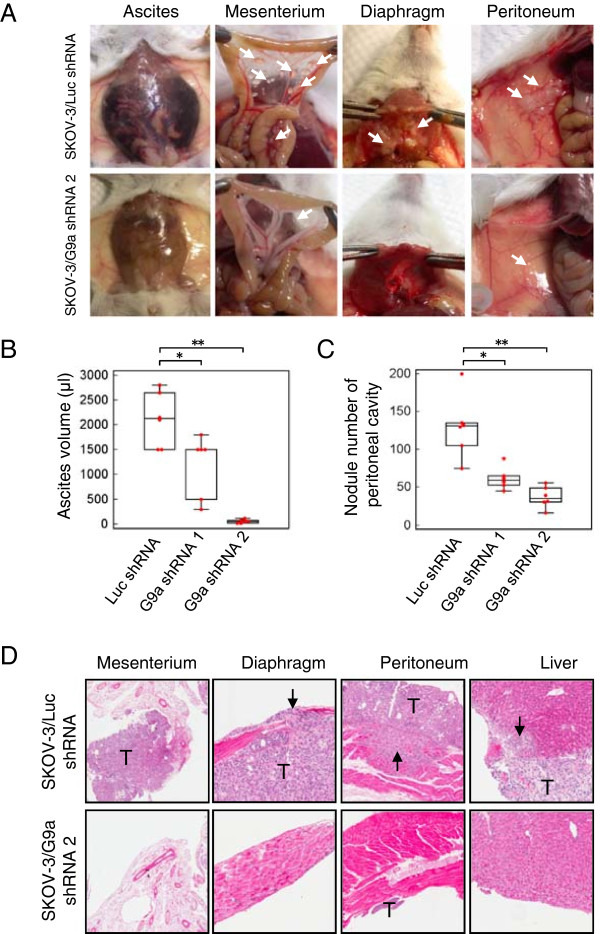
**Knockdown of G9a inhibits peritoneal dissemination of OCa cells** ***in vivo*** **. A**. SKOV-3 cells (1 × 10^6^) were suspended in 100 μl PBS and intraperitoneally injected into severe combined immunodeficient (SCID) mice (6 mice/group). After 10 weeks of inoculation, the tumor burden and ascites formation in mice receiving control or G9a knockdown cells were estimated. Representative photos of tumor formation at the mesentery, diaphragm, peritoneum, and ascites are shown. Arrows: disseminated tumors. **B**. Box and whisker plot of the ascites volumes collected from the control and G9a knock down groups. **C**. Box and whisker plot of disseminated tumors in the abdominal cavities of the control and G9a knock down groups. **D**. The histological appearance of disseminated tumors in the abdominal cavities as indicated was assayed by H & E staining tissue from mice in the control and G9a knock down groups. T: tumor, arrows: invaded tumor mass. *P < 0.05, **P < 0.01, indicate a statistically significance by one way ANOVA followed by Bonferroni test.

We next evaluated the in vivo effect of G9a knockdown on OCa metastasis. For these experiments, SKOV-3 cells that stably express G9a shRNAs (SKOV-3/G9a shRNA1 or SKOV-3/G9a shRNA2) or control shRNA were i.p. injected into cohorts of severe combined immunodeficient (SCID) mice. Mice injected with control SKOV-3 cells exhibited remarkable ascites formation (Figure 3A, left panel) ten weeks post-inoculation. The ascites volume of mice bearing SKOV-3/G9a shRNA1 or SKOV-3/G9a shRNA2 tumors was significantly lower than that of the SKOV-3/Luc shRNA group (Figure 3B). Mice injected with control SKOV-3 cells also developed tumors in the peritoneum, mesentery, diaphragm, kidney and liver. In contrast, SKOV-3/G9a shRNA1 and SKOV-3/G9a shRNA2 cells only developed small tumors with less organ involvement in the abdominal cavity (Figure 3A and C). Histological analysis of the xenografts revealed that tumors from the control groups invaded into the surrounding tissues at dissemination sites including the mesentery, diaphragm, peritoneum and liver (Figure 3D, arrows). Such invasive behavior was reduced in the tumors derived from SKOV-3/G9a shRNA1 and SKOV-3/G9a shRNA2 cells. Comparison of the number of tumor nodules, the incidence of ascites, and the amount of tumor dissemination in the abdominal cavity between the control and G9a knockdown groups is summarized in Additional file 1: Table S2. Collectively, these in vivo observations suggest that G9a is essential for enabling peritoneal metastasis of OCa cells.

### Identification of G9a-regulated genes

The ability of G9a to promote multiple steps in the progression and metastasis of OCa might be derived from its ability to pleiotropically regulate genes that are involved in diverse aspects of metastatic dissemination. In an attempt to elucidate this possibility, we performed Affymetrix HG-U133A GeneChip array analysis on SKOV-3/Luc shRNA, SKOV-3/G9a shRNA1 and SKOV-3/G9a shRNA2 cells. Unsupervised clustering analysis identified two groups of genes that significantly changed expression (>2-fold) after G9a depletion with either shRNA, including 365 downregulated genes and 234 upregulated genes. Gene Ontology (GO) analysis of biological processes suggested gene categories related to cell adhesion, migration, and protein dephosphorylation were highly altered by G9a depletion. Molecular functions analysis suggested that gene categories related to phosphatase activity, protein binding, and kinase activity were also highly altered by G9a depletion (Additional file 1: Table S3). Similarly, Ingenuity Pathway Analysis (IPA) primarily assigned the functions of cell signaling, cell morphology, cellular movement, and cell-to-cell signaling and interaction to the G9a gene signature. Moreover, IPA indicated a significant association between the G9a-regulated gene expression profile and cancer, genetic disorders, connective tissue disorders and metabolic disease (Additional file 1: Table S4).

### G9a regulates metastasis-related genes in OCa

**Figure 4:**
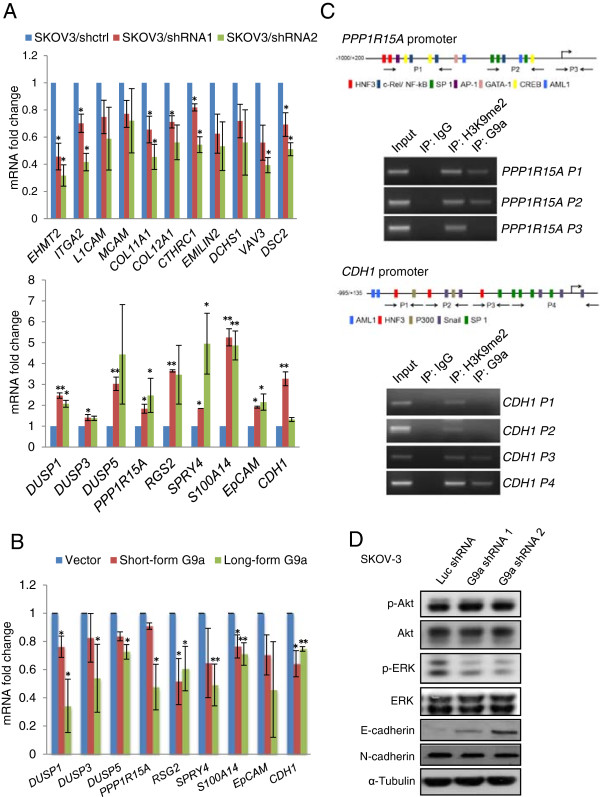
**G9a knockdown decreases metastasis-related signaling. A**. Quantitative RT–PCR verification of the expression of genes identified in the microarray: control shRNA (blue) versus G9a shRNA1- and shRNA2-expressing cells (red and green, respectively). Upper panel: genes down-regulated after G9a depletion; Lower panel: genes up-regulated after G9a depletion. Genes analyzed shown on x-axis. **B**. OV-90 cells transfected with long-form or short-form G9a for 48 h and harvested for RNA extraction. G9a-regulated genes identified from microarray as indicated were examined by quantitative RT–PCR. **C**. G9a binding and H3K9 dimethylation regions of the promoters determined by ChIP assay in SKOV-3 cells. Schematic diagram of the human *PPP1R15A* promoter (GenBank accession number NC_000019) (Top) and *CDH1* promoter (GenBank accession number NC_000016) (Bottom) shown. Numbered arrows indicate primer sets used for ChIP analysis. **D**. Akt and ERK phosphorylation status and E-/N-cadherin expression examined in control and G9a knock down cells. Results of quantitative RT–PCR are mean values of three independently repeated experiments done in duplicate and are displayed as mean ± SD. *P < 0.05, **P < 0.01, indicate a statistically significance by one way ANOVA followed by Bonferroni test.

We next used quantitative RT-PCR to confirm G9a-dependent expression of genes identified in the microarray analysis (Figure 4A). Expression of many of the genes known to have important functions in promoting tumor metastasis was found to be regulated by G9a. Vav3 oncogene (*VAV3*), a Rho guanine nucleotide exchange factor known to induce cell invasion and metastasis [24, 25], was suppressed by G9a depletion. Knockdown of G9a also blocked the expression of extracellular matrixes (ECM) and adhesion molecules that are typically expressed in invasive cancers to promote cell adhesion [26–29]. These genes include the ECM protein collagens (*COL11A1* and *COL12A1*), ECM glycoprotein (*EMILIN2*), collagen triple helix repeat containing 1 (*CTHRC1*), the cell-ECM-interaction protein α2 integrin (*ITGA2*), L1-cell adhesion molecule (*L1CAM*), melanoma adhesion molecule (*MCAM*), fibroblast cadherin 1 (*DCHS1*), and desmosomal glycoprotein 2 (*DSC2*) (Figure 4A, upper panel). On the other hand, the genes upregulated after G9a depletion include several tumor suppressor genes like dual specificity phosphatases (*DUSP1*, *DUSP3* and *DUSP5*), which mediate MAP kinase dephosphorylation and are commonly repressed in many cancer cells [30, 31]. Other potential tumor suppressor genes downregulated by G9a were GADD34 (*PPP1R15A*), a growth arrest and DNA damage-induced protein [32] and Sprouty4 (*SPRY4*), an inhibitor of the Ras/MAPK signaling cascade [33]. G9a depletion also increased the expression of two prognostic markers, RGS2 and S100A14, which have both been reported to be downregulated in metastatic tumors [34–36]. Since G9a mediated H3K9 di-methylation is known to be a major contributor in gene silencing, genes upregulated upon G9a-depletion were considered more likely to be directly regulated by G9a. Indeed, *EPCAM* and *CDH1*, both previously found to be epigenetically suppressed by G9a [14, 21], were up-regulated after G9a depletion in SKOV-3 cells. Expression of most of these identified G9a-regulated genes also increased after treatment with a G9a specific inhibitor, UNC0638 (Additional file 1: Figure S5). Furthermore, over-expression of G9a-L or G9a-S in OV-90 cells also decreased expression of these identified genes (Figure 4B). By doing ChIP assays, we also confirmed the direct binding of G9a to *CDH1*, *PPP1R15A* and *SPRY4* promoter regions in SKOV-3 cells (Figure 4C and Additional file 1: Figure S6A). However, indirect gene regulation by G9a may also exist since G9a occupancy on *DUSP5* promoter was not observed in all regions examined (Additional file 1: Figure S6B).

**Figure 5:**
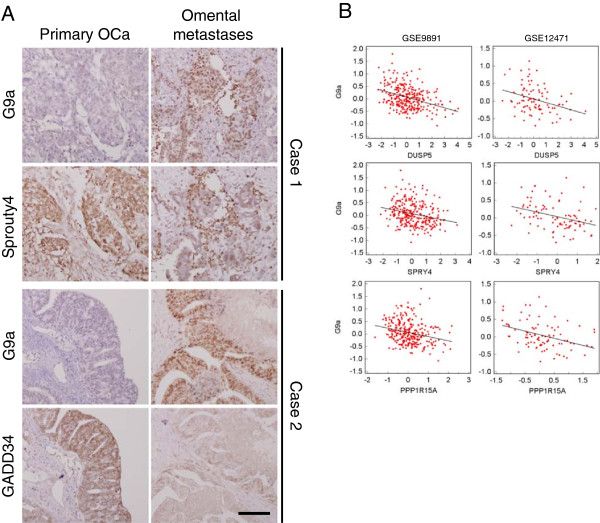
**G9a expressions clinically correlate with metastasis-related genes. A**. Representative microphotographs of immunohistochemical staining of G9a, GADD34, and Sprouty4 in matched specimens of primary ovarian tumors and metastases. Scale bar: 100 μm. **B**. mRNA expression scatter diagrams of *G9a* versus *DUSP5*, *SPRY4* or *PPP1R15A*. Red dots represent expression of indicate genes from specimens in a dataset; regression lines shown on plot. The three left plots were made from GSE9891, and the three right plots were made from GSE12471.

Finally, we examined the protein expression of G9a and G9a-regulated genes in clinical samples. G9a showed a reciprocal expression pattern with Sprouty4, GADD34 and E-cadhesin in tissues from primary ovarian tumors and omental metastases (Figure 5A and Additional file 1: Figure S7). The expression profiles of G9a and G9a-regulated genes (*DUSP5*, *SPRY4* and *PPP1R15A*) were also inversely correlated with each other in two independent datasets (Figure 4B and Additional file 1: Table S5). Consistent with the expression pattern of *DUSP5* and *SPRY4*, we found that ERK phosphorylation was significantly decreased after G9a depletion (Figure 4D). Re-expression of E-cadherin was also confirmed in the SKOV-3/G9a shRNA cells. Of note, knockdown of E-cadherin expression in SKOV-3/G9a shRNA cells rescued the invasion ability of the cells (Additional file 1: Figure S8). Taken together, these findings suggest that G9a may regulate OCa peritoneal metastasis through the regulation of metastatic genes.

## Discussion

Although increased G9a expression has previously been found in a variety of cancer tissues as compared to their normal counterparts [20, 37], the clinical significance of G9a expression in tumors has rarely been studied. Here, we observed that G9a expression was highly correlated with stage of OCa. G9a was also highly expressed in serous type OCa, the most abundant and malignant subtype of OCa. Furthermore, we demonstrated that G9a expression was markedly increased in metastatic OCa in comparison to primary ovarian tumors. Notably, G9a expression significantly correlated with shorter survival of OCa patients. In this regard, G9a expression analysis may represent a novel prognostic marker of OCa.

OCa progression usually results in peritoneal metastasis, which is the most common cause of morbidity and mortality in OCa patients. Peritoneal metastasis is a complex process that involves detachment, ascitic current, immune evasion, spheroid formation, pro-invasive ascitic components, ascites formation, implantation and invasion [2]. To assess the biological significance of increased G9a expression in OCa cells, we suppressed G9a expression in highly metastatic OCa cell lines and performed in vitro and in vivo functional assays. Anoikis assays may partially reflect the environment that cancer cells encounter in the ascites fluid. Cancer cells must survive and grow into spheroids before spreading into the abdominal cavity. Our data from anoikis assays and soft agar assays revealed the importance of G9a in promoting anoikis resistance and anchorage-independent growth. In vitro adhesion assays also demonstrated a defect in OCa cell adhesion after G9a depletion. The mobility assays suggest that the migration and invasion of OCa cells into the peritoneum after implantation may also be affected in the absence of G9a expression. Since cell adhesion is the first step of cell invasion, we cannot exclude the possibility that G9a suppresses cell invasion through adhesion-inhibition. However, we only performed adhesion assays that used a very short time period (1 h). During the wound healing assay, we also observed complete adherence 18 h after cell seeding. Therefore, the effect of G9a on invasion-suppression may be caused by synergistic inhibition of cell adhesion and cell migration.

We have used long- and short-form G9a overexpression models in the migration, invasion and adhesion assays. In these functional assays, our results show that long-form G9a overexpression significantly increased cell migration, invasion and HMC adhesion of OV-90 cells, while short-form G9a only significantly increased cell invasion of OV-90 cells. In contrast, overexpression of either long-form or short-form G9a in SKOV-3 cells almost completely restored diminished cell invasion and adhesion caused by G9a depletion. In these experiments, overexpression of G9a only resulted in a 1.2 ~ 1.5 fold increase in cell migration/invasion/adhesion, while knockdown of G9a resulted in a more than 50% decrease in cell migration/invasion/adhesion. These results may indicate that overexpression of G9a proteins is essential but not fully sufficient to promote cell migration, invasion or adhesion of ovarian cancer cells. Indeed, fully functional G9a requires complex formation with GLP and/or WIZ proteins [38, 39]. Since G9a does not have a DNA-binding domain, its epigenetic regulation of genes requires cooperation with DNA-binding proteins like transcription factors. OV-90 cells may also lack other components for fully functional G9a-protein complex formation. Therefore, in OV-90 cells, overexpression of either long- or short-form G9a, alone, did not result in significant change in all cellular functions examined. Otherwise, re-expression of G9a in highly adhesive/invasive cells such as SKOV-3 cells would be expected to quickly re-constitute the functional G9a-protein complex and rescue cellular functions originally blocked by G9a knockdown.

Consistent with our in vitro findings, G9a knockdown cells also developed fewer tumors in the abdominal cavity than control cells in our animal models, which may be due to a decrease in their adhesiveness or anoikis resistance. G9a knockdown cells also grew smaller tumors, which was in keeping with our observation in in vitro tumorigenesis assay. Decreased migration and invasion of G9a knockdown cells in vitro was also consistent with the fewer invasions observed in vivo. Collectively, we observed an attenuated ability of G9a-deficient OCa cells to metastasize in a peritoneal dissemination model and, therefore, propose that the effect of G9a on cellular anoikis, adhesion and mobility may be relevant to the development of peritoneal metastases of OCa cells.

Several G9a-regulated genes, including *MASPIN*, *DSC3*, *EpCAM* and *CDH1*, have been previously identified [13, 14]. The majority of these genes behave as tumor suppressors in different cancers. Recently, a microarray analysis of the effect of G9a knockdown in breast cancer identified a cohort of G9a-regulated genes involved in epithelial-mesenchymal transition (EMT), a phenotypic conversion linked with metastasis [14]. In their study, the authors found that epithelial markers such as claudins and E-cadherin were upregulated after G9a depletion, whereas mesenchymal markers, including N-cadherin and vimentin, were downregulated. Consistent with their findings, we also observed E-cadherin upregulation after G9a depletion in our microarray, Q-PCR and immunoblot analyses. Functionally, E-cadherin expression was also important for OCa peritoneal metastasis [40], and we observed a rescue effect of E-cadherin shRNAs on the invasiveness of G9a knockdown SKOV-3 cells (Additional file 1: Figure S8). However, other EMT markers, including N-cadherin, remained unchanged in G9a knockdown OCa cells in our study. These findings suggest that G9a may differentially regulate gene expression in different cell contexts.

The identification of several G9a-regulated genes in our study confirmed the potential pro-metastatic role of this histone methyltransferase. We first noticed that G9a suppressed the expression of innate negative regulatory proteins of mitogen-activated protein kinases (MAPKs), including dual-specific phosphatase family proteins (DUSPs) and Sprouty4. G9a suppressed the ERK-specific phosphatase DUSP5 to an even greater extent. Sprouty4, an inhibitor of Ras/MAPK signaling that exerts its suppression effect through Raf1 binding [33], may also result in ERK inhibition in OCa. Indeed, ERK activation was dramatically suppressed after G9a depletion. There are high frequencies of KRAS or BRAF mutations found in OCa and their mutation status significantly correlates with ERK activation [41, 42]. A recent phase II study of a MEK1/2 inhibitor, Selumetinib, has shown a total clinical benefit rate of 80% in OCa [43]. Unexpectedly, the therapeutic response did not correlate with KRAS or BRAF mutations. 35% of responders had neither BRAF nor KRAS mutations. It would be of interest to determine whether G9a expression levels might serve as a biomarker for predicting therapeutic response to Selumetinib or other MEK1/2 inhibitors in OCa. GADD34, a stress-response inhibitor of cell growth, is believed to induce apoptosis or growth arrest in tumors [44]. Although we did not observe a defect in cell proliferation after G9a knockdown under normal culture conditions, anoikis and soft agar assays demonstrated a decrease in viability. Long-term culture conditions such as soft agar assays or xenograft experiments cannot exclude the existence of proliferation disadvantages in cells with G9a depletion. The reciprocal mRNA expression patterns of *G9a* versus *DUSP5*, *SPRY4*, *PPP1R15A* in datasets as well as the protein expression patterns of G9a versus Sprouty4 and GADD34 in clinical samples strengthen the correlation between G9a and these tumor suppressive genes in OCa progression.

Collectively, our findings suggest that G9a is a “pro-metastatic” HMT, and this HMT acts at multiple steps in the OCa progression and metastasis cascade by regulating a cohort of specific genes. Advanced metastatic OCa is a fatal phase of the disease that has only palliative therapeutic options, and it is a disease in urgent need of new diagnostic and therapeutic strategies. Due to the tremendous molecular complexity of advanced OCa, new therapeutic strategies are being envisioned that would disable multiple networks of tumor maintenance, rather than individual signaling pathways. Here we have identified a G9a regulatory network that plays a pivotal role in the progression and metastasis of OCa by affecting an array of effectors. Therefore, we envision that G9a may be an attractive target for therapeutic intervention.

## Materials and methods

### Patients and samples

Tissue blocks with samples from 208 patients who were diagnosed with Federation Internatonale des Gynaecologistes et Obstetristes (FIGO) stage I to Stage IV advanced epithelial OCa and had undergone debulking surgery at the National Taiwan University Hospital and the Taipei Veterans General Hospital from 2001 to 2007 were obtained. None of the patients had received pre-operative adjuvant chemotherapy or radiation therapy. Approval for the study was obtained from the ethics committee of each hospital. Gene expression profiles were obtained from http://www.ncbi.nlm.nih.gov/geo/ [45–50]. The probe sets of G9a are 202326_at or 207484_s_at.

### Immunohistochemistry

A scoring system was devised to assign a staining intensity score for G9a expression from 0 (no expression) to 3 (highest intensity staining). Immunostaining was classified as either low or high expression according to both intensity and extent. Low expression was defined as either no staining present (staining intensity score = 0) or positive staining present in less than or equal to 20% of the cells (staining intensity score = 1). High expression was defined as either positive staining present in 20%–50% of the cells (staining intensity score = 2) or more than 50% of the cells (staining intensity score =3).

### Cell lines and cell culture

The human OCa cell lines were obtained from the American Type Culture Collection (Rockville, MD). All of the cell lines were cultured according to ATCC’s propagation protocols. Primary ovarian surface epithelial (OSE) cells were isolated from ovarian biopsies from women with nonmalignant gynecological disease as previously described [51]. Human adult mesothelial cells (Zenbio, Cat: F-MES-F) were cultured using media MSO-1 (Zenbio) according to the manufacturer’s guidelines.

### Western blot analysis

Western blot analysis was carried out as previously described [21] and using the following primary antibodies: anti-G9a (3306, Cell Signaling Technology), phospho-Akt1 (sc-33437, Santa Cruz), Akt1 (sc-8312, Santa Cruz), phospho-ERK1/2 (sc-16982, Santa Cruz), ERK1 (sc-93, Santa Cruz), E-cadherin (ab1416, Abcam), N-cadherin (610921, BD Transduction Laboratory), and α-tubulin (T5168, Sigma-Aldrich).

### Lentivirus infection

The G9a shRNAs were purchased from the National RNAi core Facility at Academia Sinica in Taipei, Taiwan. The target sequences of G9a shRNA 1, G9a shRNA 2, G9a shRNA 3’UTR, and Luciferase shRNA were 5’-CAC ACA TTC CTG ACC AGA GAT-3’, 5’-GCT CCA GGA ATT TAA CAA GAT-3’, 5’-CAC ACA TTC CTG ACC AGA GAT-3’ and 5’-GCG GTT GCC AAG AGG TTC CAT-3’, respectively. Knockdown of G9a expression was accomplished by lentivirus infection and 2 μg/ml puromycin was used to select cells with stable knockdown.

### Anoikis assay

Anoikis resistance was detected by seeding 5 × 10^4^ cells in ultra-low attachment plates (Corning). After 24 h of culture, the cells were resuspended in 0.4% trypan blue (Sigma), and cell viability was assessed.

### Wound-healing migration assay

Cells were seeded in culture media on 24-well plates at a density of 1.2 × 10^5^ cells per well for SKOV-3 cells and 3 × 10^5^ cells per well for ES-2 cells. The confluent monolayer of cells was scratched with a fine pipette tip, and cell migration into the wound was visualized and scored by measuring the size of the initial wound and comparing it to the size of the wound after 24 h by microscopy.

### Invasion assay

Cells were seeded onto the upper chambers of Matrigel-coated 24-well invasion inserts of 8 μm pore membranes (BD Biosciences) in culture media at a density of 4 × 10^4^ (SKOV-3) or 2 × 10^4^ (ES-2) cells/well and 1 ml of the same media was placed in the lower chamber. After 24 h, the cells were fixed and cells on the upper side of the filters were removed with cotton-tipped swabs. Cells on the underside of the filters were stained with crystal violet and counted under a microscope (type 090–135.001, Leica Microsystems, Wetzlar, Germany).

### Human mesothelial cell adhesion assay

HMCs were seeded in Collagen I-coated 24-well plate and allowed to grow to 100% confluence. CellTracker™ Green CMFDA (Molecular Probes, Eugene, OR) labeled OCa cells were overlaid on the HMC monolayer, and the plate was incubated at 37°C for 60 min. After washing, the cells that were adherent to the HMC monolayer were counted under a microscope.

### Peritoneal metastasis model

Animal experiments were performed in accordance with a protocol approved by the NTUCM and the NTUCPH Institutional Animal Care and Use Committee. Six age-matched NOD/SCID female mice (6 weeks old) were used for each group. Cells (1 × 10^6^) were re-suspended in 0.1 ml PBS and injected into the abdominal cavity. Ascites formation and body weight were measured once a week. The mice were sacrificed after 10 weeks, and the volume of ascites and quantity of nodules present in the abdominal cavity of each mouse was measured.

### Cell line RNA preparation and microarray analysis

RNA was isolated from cells using TRIzol (Invitrogen), purified on RNeasy columns (Qiagen), and checked for integrity by Agilent testing. cDNA was generated and hybridized to Human Genome U133 Plus 2.0 Arrays (Affymetrix) according to the manufacturer’s instructions. The microarray data sets have been deposited in Gene Expression Omnibus as GSE41226. The 234 up and 365 down regulated gene (fold changed >2) are listed as Additional file 2: Table S7.

### Quantitative reverse transcription-PCR

cDNA was synthesized from 2 μg Trizol-extracted RNA of each sample using the SuperScript III first strand synthesis kit (Invitrogen) according to the manufacturer’s instructions. Quantitative reverse transcription-PCR was carried out using the SYBR green quantitative PCR master mix (Fermentas) and Bio-Rad iQ5 detection system. Forward and reverse primers are shown in Additional file 1: Table S6. Gene expression values were calculated relative to GAPDH expression for each sample.

### Chromatin immunoprecipitation assay

Chromatin immunoprecipitation (ChIP) assays were performed according to the manufacturer’s protocol (Upstate). The chromatins were incubated with 4 μg each of anti-K9 dimethylated histone H3 and anti-G9a (Upstate) at 4°C overnight. Immunoprecipitated DNA was analyzed by quantitative PCR by using specific primers as described in Additional file 1: Table S5.

### Statistical analysis

Statistical analyses of clinicopathological data were performed using the chi-square exact test. Survival curves were obtained using the Kaplan–Meier method. Cox proportional hazards regression was used to test the prognostic significance of factors in univariate and multivariate models. The association between G9a expression level and metastasis in paired specimens was analyzed using a paired *t*-test and displayed by box-plot. One way ANOVA followed by Bonferroni tests were used to compare data between groups in the in vitro functional assays and in vivo animal experiments. *P* < 0.05 was considered statistically significant.

## Consent

Written informed consent was obtained from the patient for the publication of this report and any accompanying images.

## Electronic supplementary material

Additional file 1: Figure S1-S8, Table S1-S6: Additional evidences support that G9a is a marker of aggressive ovarian cancer that promotes peritoneal metastasis. (PDF 720 KB)

Additional file 2: Table S7: G9a regulated gene list discovered by microarray analysis in SKOV-3 cells. (XLSX 140 KB)
