# Supplementary material for: The H3K9 methyltransferase G9a is a marker of aggressive ovarian cancer that promotes peritoneal metastasis
Source: Mol Cancer. 2014 Aug 12;13:189. doi: 10.1186/1476-4598-13-189 (PMC4260797; doi:10.1186/1476-4598-13-189)

Supplemental Information

Title: The H3K9 methyltransferase G9a is a marker of aggressive ovarian cancer that promotes peritoneal metastasis

Kuo-Tai Hua, Ming-Yang Wang, Min-Wei Chen, Lin-Hung Wei, Chi-Kuan Chen, Ching-Huai Ko, Yung-Ming Jeng, Pi-Lin Sung, Yi-Hua Jan, Michael Hsiao, Min-Liang Kuo, Men-Luh Yen

Correspondence: Dr. Min-Liang Kuo (kuominliang@ntu.edu.tw) and Dr. Men-Luh Yen (mlyen@ntu.edu.tw)

Supplemental materials and methods

In vitro cell growth assay

Control shRNA, G9a shRNA 1 and shRNA 2 cells were added to 96-well dishes initially containing 5×10^3 cells per well. At regular intervals, the growth rates of G9a knockdown cells were determined using MTT (Bio-Rad) as a substrate. The principle of the MTT assay relies on the activity of mitochondrial dehydrogenases, which reduce the water-soluble tetrazolium salt to form a purple insoluble formazan product. The amount of MTT formazan product was analyzed spectrophotometrically at a wavelength of 570 nm. Each individual experiment was repeated at least three times.

Colony-forming assay in soft agar

Colony formation in soft agar was assessed. Briefly, 2 mL of mixture of serum supplemented medium and 0.5% agar at 40°C were added in a 35-mm culture dish and allowed to solidify to form the agar base layer. Next, on top of the base layer was added a mixture of serum supplemented medium and 0.35% agar (total of 2 mL) containing 5,000 cells at 40°C and allowed to solidify to form the top agar. Subsequently, the dishes were kept in a tissue culture incubator maintained at 37°C and 5% CO_2 for 14 days to allow for colony growth. All assays were done in triplicate. The colony assay was terminated at day 14, and total colonies were counted under microscopy.

In vitro adhesion assay

Lentivirus-infected SKOV-3 or ES-2 cells were fluorescently labeled with CellTracker™ Green CMFDA (green; Molecular Probes, Eugene, OR). After recovery for 30 min, 2.5×10^5 (for the SKOV-3 cells) or 4×10^5 cells/well (for the ES-2 cells) were placed in a 96-well plate that was pre-coated with collagen type 1 (50 μg/ml), fibronectin (5 μg/ml), or Matrigel (20 μg/ml). After incubation for 1 h at 37 ℃, the cells were washed and fixed. The number of adhesive cells was quantified by measuring the fluorescent intensity (excitation, 590 nm; emission, 620 nm) with a fluorescence spectrophotometer (VICTOR2 Multilabel Counter, PerkinElmer Life And Analytical Sciences, Inc.)

Supplemental figures and figure legends

Figure S1. G9a depletion did not affect cell proliferation in short-term culture conditions.

The cell growth effect of G9a shRNAs and control shRNA in SKOV-3 and ES-2 cells. The

proliferation rates of G9a stable knockdown cells were determined by MTT assay. The results are mean values of three independently repeated experiments done in quadruplicate and are displayed as mean ± SD.

Figure S2. G9a depletion inhibits anchorage-independent growth.
Colony assays in soft agar were done using SKOV-3 (4000 cells/well) and ES-2 cells (2000 cells/well) infected with G9a shRNAs or control shRNA. Representative images of the colonies in soft agar 2 weeks after initial seeding are shown. Total colonies in the well were counted. Data shown are means ± SD of three independent experiments. Scale bars: 500 μm for ES-2 cells and 200 μm for SKOV-3 cells. *$P<0.05$, indicate a statistically significance by one way ANOVA followed by Bonferroni test.

Figure S3. G9a depletion suppresses OCa cell adhesion.
A. Adhesion of G9a knockdown cells to ECM-coated surfaces. Cells were labeled with CellTracker™ Green CMFDA fluorescent dye and plated onto 96-well plates pre-coated with fibronectin, type I collagen, or Matrigel. After incubation at 37℃ for 1 h, the plates were washed and fluorescence intensity was detected using a fluorescence spectrophotometer. B. SKOV-3 cells infected with G9a shRNAs or control shRNA were labeled with CellTracker™ Green CMFDA, plated onto Human primary mesothelial cells (HMCs) monolayer, and incubated at 37℃ for 1 h. The plates were then washed and fluorescence cells were counted under fluorescence microscope. Representative photos shown above the plot. Scale bar, 100 μm. Three independent experiments were performed in triplicate, and the results are presented as means ± SD. *$P<0.05$, **$P<0.01$, indicate a statistically significance by one way ANOVA followed by Bonferroni test.

Figure S4. G9a over-expression promotes cell adhesion and cell motility in OV-90 cells.
A. Protein expression of overexpressed long-form and short-form G9a in OV-90 cells. B. Migration and invasion of long-form or short-form G9a overexpressed OV-90 cells. Wound-healing assays and transwell invasion assays were used to determine the amount of cell migration and invasion. C. Long-form or short-form G9a overexpressed OV-90 cells were labeled with CellTracker™ Green CMFDA, plated onto HMCs monolayer, and incubated at 37℃ for 1 h. The plates were then washed and fluorescence cells were counted under fluorescence microscope. Representative photos shown above the plot. Scale bar, 100 μm. Data shown are means ± SD of three independent experiments. *$P<0.05$, **$P<0.01$, indicate a statistically significance by one way ANOVA followed by Bonferroni test.

Figure S5. UNC0638 suppresses invasion and induces expression of G9a-suppressed genes in SKOV-3 cells.

A. SKOV-3 cells were treated with 1 and 3μM of the G9a inhibitor, UNC0638 (U4885, Sigma-Aldrich), for 24h and harvested for protein extraction and subjected to SDS-PAGE. H3K9me2 levels were examined by western blotting and specific antibody. B. Transwell invasion assays were performed in SKOV-3 cells in the presence of 1 and 3μM UNC0638. Cells invaded into opposite site of transwell were counted and presented as % of control. Scale bar: 100 μm. C. RNA were harvested from SKOV-3 cells treated with DMSO or 3μM UNC0638 for 24h. G9a-regulated genes identified from microarray as indicated were examined by quantitative RT–PCR. Data shown are means ± SD of three independent experiments. **P*<0.05, ***P*<0.01, indicate a statistically significance by one way ANOVA followed by Bonferroni test.

Figure S6. Occupancies of G9a on promoter regions of *SPRY4* and *DUSP5*.

G9a binding and H3K9 dimethylation regions of the promoters were determined by ChIP assay in SKOV-3 cells. Schematic diagram of the human *SPRY4* promoter (GenBank accession number NC_000005) (A) and *DUSP5* promoter (GenBank accession number NC_000010) (B) are shown. Numbered arrows indicate the primer sets used in the ChIP analysis.

Figure S7. G9a and E-cadherin expression inversely correlated with each other.

Representative microphotographs of immunohistochemical staining of G9a and E-cadherin in matched specimens of primary ovarian tumors and metastases. Scale bars: 100 μm.

Figure S8. Knockdown of E-cadherin rescues G9a-depletion-induced cell invasion.

Control and G9a stable knockdown SKOV-3 cells were infected with lentiviruses carrying either control or CDH1 shRNA for 48h and subjected to transwell invasion assays. Knockdown efficiencies were examined by western blotting. Cells invaded into opposite site of transwell were counted and presented as % of control. Data shown are means ± SD of three independent experiments. **P*<0.05, indicate a statistically significance by one way ANOVA followed by Bonferroni test.

Supplemental tables

Table S1. Clinicopathologic characteristics of patients with associated expression of G9a protein in ovarian cancer.

Characteristic	Low expression (score 0-1)	High expression (score 2-3)	*P*-value
Age (mean±SD) (in years)	51.75±11.48	54.79±12.33	
FIGO stage, no. of patients			
I-II	60	30	0.002*
III-IV	52	66	
Histological subtype, no. of patients			
Serous type	40	50	0.025*
Non-serous type	72	46	
Histological grade, no. of patients			
G1	13	8	0.037*
G2	25	14	
G3	35	49	

- *P*-values were derived with Chi-square tests.

Table S2. Comparison of metastatic behavior of animals following i.p. injection of SKOV-3 cells.

	Nodules formation	Ascites	Disseminated metastasis				
			Mesenterium	Peritoneum	Diaphragm	Liver	Kidney
SKOV-3/Luc shRNA	130 ± 41	6/6	6/6	6/6	6/6	2/6	2/6
SKOV-3/G9a shRNA 1	62 ± 15	4/6	6/6	5/6	5/6	0/6	1/6
SKOV-3/G9a shRNA 2	38 ± 14	0/6	6/6	5/6	1/6	0/6	0/6

- Ascites formation: > 0.5 mL of ascitic fluid

Table S3. Gene Ontology analysis identifies the biological processes and molecular functions significantly associated with G9a-regulated genes. (Top 20 categories obtained in each analysis shown.)

Category	Rank	Term	Genes	%	*P-Value*
GOTERM_BP	1	regulation of cell adhesion	17	3.7	1.40E-07
	2	positive regulation of cell adhesion	11	2.4	1.20E-06
	3	protein amino acid dephosphorylation	15	3.3	3.00E-06
	4	regulation of leukocyte migration	7	1.5	4.80E-06
	5	phosphate metabolic process	46	10.1	9.90E-06
	6	phosphorus metabolic process	46	10.1	9.90E-06
	7	regulation of cell migration	16	3.5	1.10E-05
	8	response to endogenous stimulus	26	5.7	1.10E-05
	9	regulation of locomotion	17	3.7	1.30E-05
	10	response to steroid hormone stimulus	17	3.7	1.30E-05
	11	regulation of cell motion	17	3.7	1.30E-05
	12	dephosphorylation	15	3.3	1.70E-05
	13	positive regulation of leukocyte migration	6	1.3	1.70E-05
	14	response to hormone stimulus	24	5.3	2.00E-05
	15	regulation of cell proliferation	39	8.6	2.00E-05
	16	protein kinase cascade	24	5.3	2.30E-05
	17	response to inorganic substance	17	3.7	2.80E-05
	18	cell migration	20	4.4	3.00E-05
	19	response to calcium ion	9	2	4.10E-05
	20	neuron differentiation	26	5.7	4.30E-05
GOTERM_MF	1	extracellular matrix binding	7	1.5	3.10E-05
	2	phosphoprotein phosphatase activity	15	3.3	3.30E-05
	3	protein tyrosine phosphatase activity	11	2.4	1.60E-04
	4	protein tyrosine/serine/threonine phosphatase activity	7	1.5	5.20E-04
	5	protein complex binding	13	2.9	2.20E-03
	6	phosphatase activity	15	3.3	2.20E-03
	7	integrin binding	7	1.5	2.50E-03
	8	growth factor binding	9	2	3.20E-03

	9	enzyme binding	23	5.1	5.80E-03
	10	heparin binding	8	1.8	1.10E-02
	11	fibronectin binding	3	0.7	1.10E-02
	12	protein tyrosine kinase activator activity	3	0.7	1.40E-02
	13	identical protein binding	25	5.5	1.50E-02
	14	cytokine activity	11	2.4	1.70E-02
	15	carbohydrate binding	16	3.5	2.00E-02
	16	kinase regulator activity	7	1.5	2.20E-02
	17	calcium ion binding	32	7	2.50E-02
	18	laminin binding	3	0.7	2.60E-02
	19	protein kinase activity	23	5.1	2.80E-02
	20	iron ion binding	14	3.1	3.00E-02

Table S4. IPA identifies the molecular and cellular functions, and the diseases and disorders, which most significantly associate with G9a regulated genes.

Category	*P*-value		Molecules
Molecular and cellular functions			
Cellular Growth and Proliferation	6.06E-08-2.13E-03	99	DLC1, PTPRR, DCHS1, DLG5, EMILIN2, etc.
Cellular Movement	4.78E-11-2.05E-03	88	S100A14, TAGLN, ITGA2, L1CAM, CITED2, etc.
Cell-To-Cell Signaling and Interaction	1.04E-06-1.72E-03	41	PPP1R15A, DOK3, ITGA2, L1CAM, etc.
Cell Morphology	6.02E-08-1.85E-03	32	RGS2, ITGA2, L1CAM, FYN, etc.
Connective Tissue Development and Function	6.8E-06-1.94E-03	32	DCHS1, CITED2, COL11A1, COL12A1, COL1A1, etc.
Cell Signaling	1.5E-04-1.11E-03	21	DAPK3, PTPN11, RGS2, ITGA2, L1CAM, VAV3, etc.
Diseases and Disorders			
Genetic Disorder	5.79E-12-1.93E-03	245	TAGLN, DLG5, PTPRM, EDIL3, EMILIN2, FNDC1, etc.
Cancer	1.78E-14-1.93E-03	161	ADAM23, ARHGDIB, RGS2, S100A14, DCHS1, VAV3, etc.
Metabolic Disease	1.93E-10-1.39E-03	124	PTPRR,TRIB3, ADAM23, DSC2, COL5A1, etc.
Connective Tissue Disorders	8.64E-11-1.39E-03	94	MCAM, THBS1, COL11A1, COL12A1, COL1A1, COL5A1, MATN2, etc.

Table S5. Expression correlations of G9a and G9a-regulated genes.

Obtained database		comparison group	Spearman's coefficient (Rho + 95%CI)	Significance level
GSE9891	(N=284)	G9a vs DUSP5	-0.330 (-0.430 to -0.222)	P<0.0001
		G9a vs SPRY4	-0.269 (-0.373 to -0.157)	P<0.0001
		G9a vs PPP1R15A	-0.289 (-0.392 to -0.179)	P<0.0001
GSE12471	(N=90)	G9a vs DUSP5	-0.331 (-0.504 to -0.133)	P=0.0018
		G9a vs SPRY4	-0.382 (-0.546 to -0.190)	P=0.0003
		G9a vs PPP1R15A	-0.408 (-0.567 to -0.219)	P=0.0001

Abbreviations: Rho, Spearman's coefficient of rank correlation; CI, confidence interval.

Table S6. Primers used in quantitative real-time RT-PCR and ChIP assays.

Primers used in quantitative real-time RT-PCR	
G9a F’	5’ ACAAAGAGGGGGACACAGCA 3’
G9a R’	5’ ATGGTGGACGTCTCGCAGTT 3’
L1CAM F'	5’ GGTACGTGTGGCCTCTCCTC 3’
L1CAM R'	5’ TACACGGTCACACCCAGCTC 3’
MCAM F'	5’ GACGAGCGCATCTTCTTGTG 3’
MCAM R'	5’ TCTGGGACGACTGAATGTGG 3’
COL11A1 F'	5’ CGTAACAACCCTCGCATTGA 3’
COL11A1 R'	5’ CTGTTTTGTTGGGGCACTGA 3’
COL12A1 F'	5’ GAACGGCACGTGTTCATTGT 3’
COL12A1 R'	5’ TAGCTGGGGAAAGACCCTGA 3’
CTHRC1 F'	5’ GTGCTTACAAGGGCCAGCAG 3’
CTHRC1 R'	5’ CACTCCGCAATTTTCCCAAG 3’
EMILIN2 F'	5’ GTACAACTGTGCCTGGAACC 3’
EMILIN2 R'	5’ GCTGGGTTCATTATCTGTGG 3’
VAV3 F'	5’ CAGAGAAACGGACCAATGGA 3’
VAV3 R'	5’ CATACCAGGGTTGGCAAGAA 3’
DSC2 F'	5’ GGGCTTCTGGGACGTCTAAA 3’
DSC2 R'	5’ GATTCCGAGGTCTGGTGTCC 3’
PPP1R15A F'	5’ ACCCCCTGCAAGTGCTTTCT 3’
PPP1R15A R'	5’ TTCTCCCCAGTCCTCAGCAG 3’
SPRY4 F'	5’ CCGGCTTCAGGATTTACACA 3’
SPRY4 R'	5’ CACATGGCTGGTCTTCACCT 3’
S100A14 F'	5’ GGGGCCAGCTATAGGACAAC 3’
S100A14 R'	5’ ACAGTTGCTCGGCATGAGAT 3’
DUSP1 F’	5’ TCCTGCCCTTTCTGTACCTG 3’
DUSP1 R’	5’ ACCCTTCCTCCAGCATTCTT 3’
DUSP3 F’	5’ GGGAAGTGATGGAACAGGAA 3’
DUSP3 R’	5’ GCCAAAAGGGGACCTTAGAG 3’
DUSP5 F'	5’ ACAGCCACACGGCTGACATT 3’
DUSP5 R'	5’ AAGCCAAAGTTGGGCGAGAC 3’
DCHS1 F'	5’ CAACCCTCCTGGCACATCTC 3’
DCHS1 R'	5’ AGATGGGCACTCCCAAAGGT 3’
EPCAM F’	5’ ATCCTGACTGCGATGAGAGC 3’

EPCAM R’	5’ ACGCGTTGTGATCTCCTTCT 3’
ARHGDIB F’	5’ AGCTGGACAGCAAGCTCAAT 3’
ARHGDIB R’	5’ AGGGCTTCCAGATCTCCAGT 3’
ITGA2 F'	5’ GAAGTCTGTTGCCTGCGATG 3’
ITGA2 R'	5’ GGAACATTCCCATCCGAAGA 3’
Primers used in ChIP assays	
PPP1R15A-P1 F’	5’ GGTGAAATCCCGTCTCAGAA 3’
PPP1R15A-P1 R’	5’ ACGCCCGGCTAATTTTGTAT 3’
PPP1R15A-P2 F’	5’ AATAAATTGGGGTGGGAAGG 3’
PPP1R15A-P2 R’	5’ GTGCTGACGTCACGAAGAGA 3’
PPP1R15A-P3 F’	5’ TCTTATGCAAGACGCTGCAC 3’
PPP1R15A-P3 R’	5’ GTTCCGACCCCTTACTCACC 3’
CDH1-P1 F’	5’ AGCGAGACTCCGTCTCAAAA 3’
CDH1-P1 R’	5’ GCGCTGTGTCTCCCTGTATT 3’
CDH1-P2 F’	5’ GCCCCGACTTGTCTCTCTAC 3’
CDH1-P2 R’	5’ TTTTGGAGATGGGGTCTCAC 3’
CDH1-P3 F’	5’ CTCCAGCTTGGGTGAAAGAG 3’
CDH1-P3 R’	5’ TAGGTGGGTTATGGGACCTG 3’
CDH1-P4 F’	5’ ACTCCAGGCTAGAGGGTCACC 3’
CDH1-P4 R’	5’ CCGCAAGCTCACAGGTGCTTTGCAGTTCC 3’

Figure S1. G9a depletion did not affect cell proliferation in short-term culture conditions.

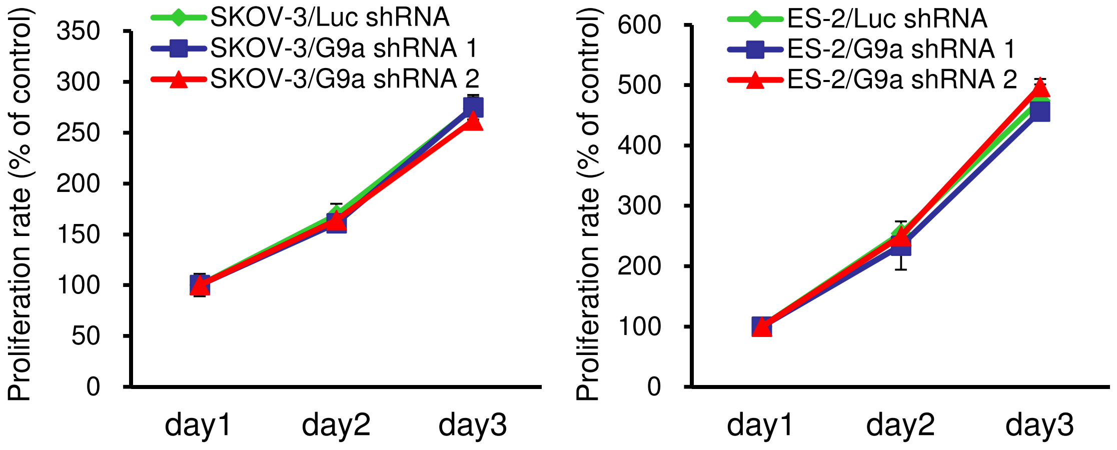

Figure S2. G9a depletion decrease anchorage-independent growth ability.

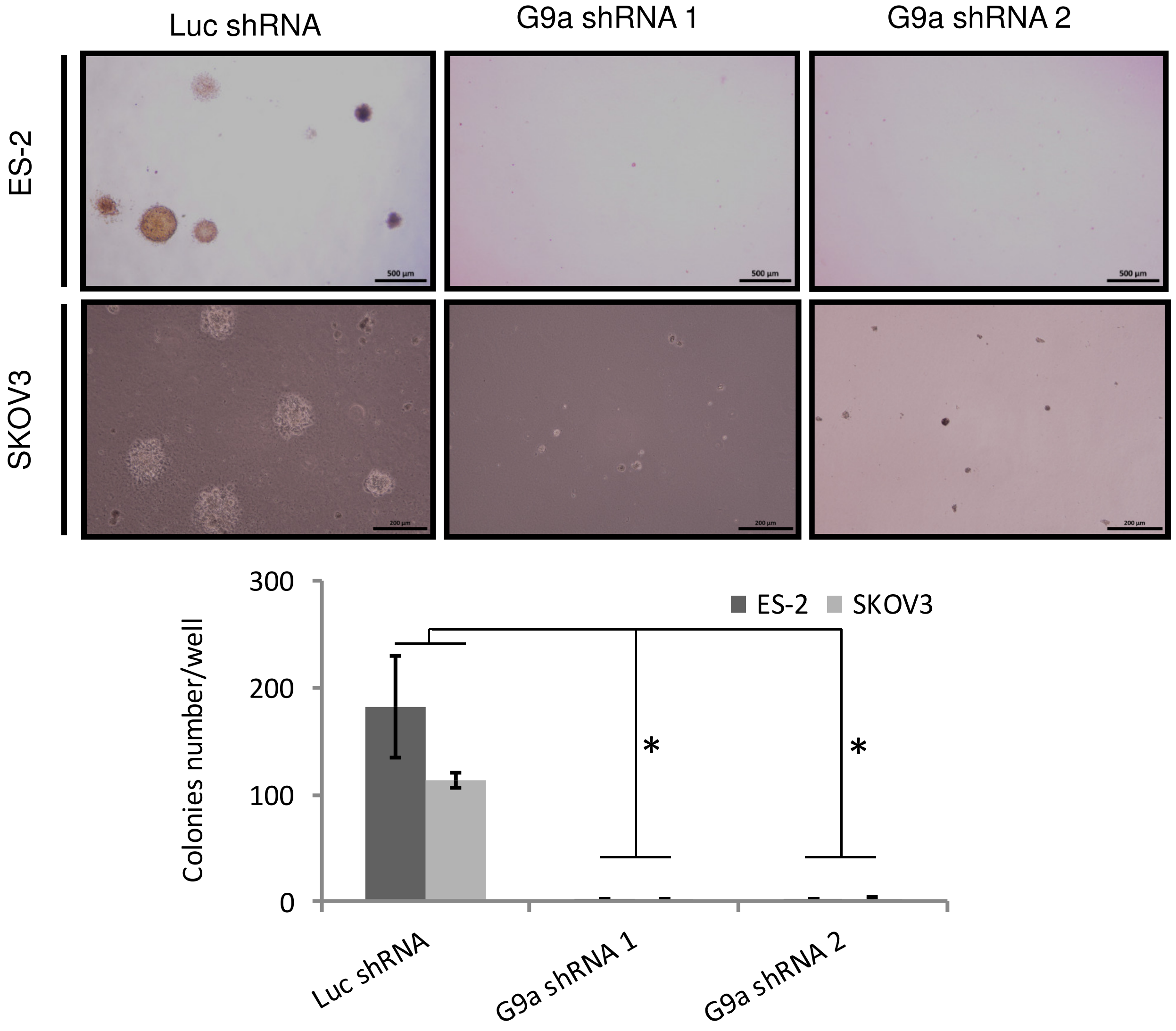

Figure S3. G9a depletion suppresses OCa cell adhesion.

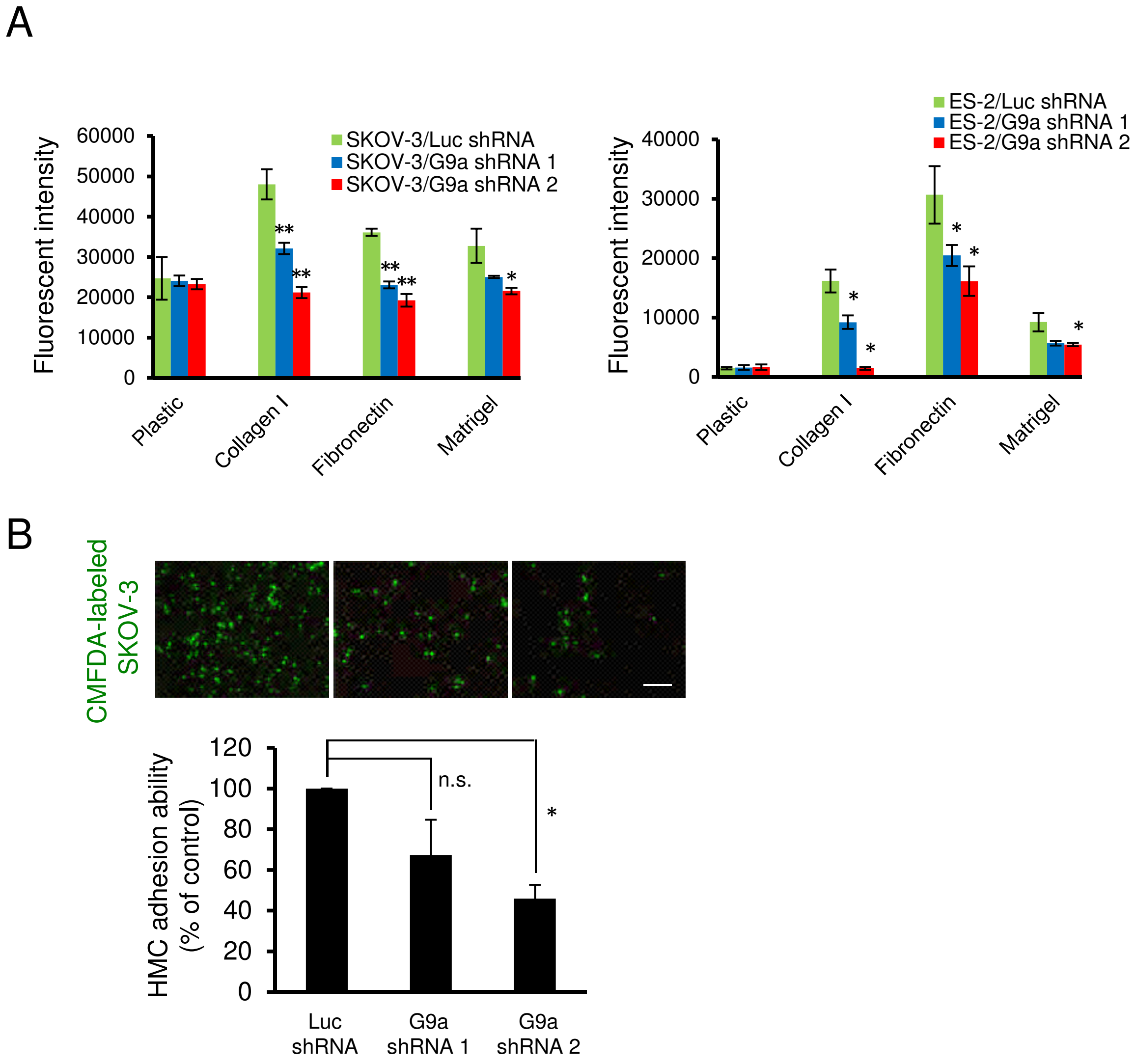

Figure S4. G9a over-expression promotes cell adhesion and cell motility in OV-90 cells.

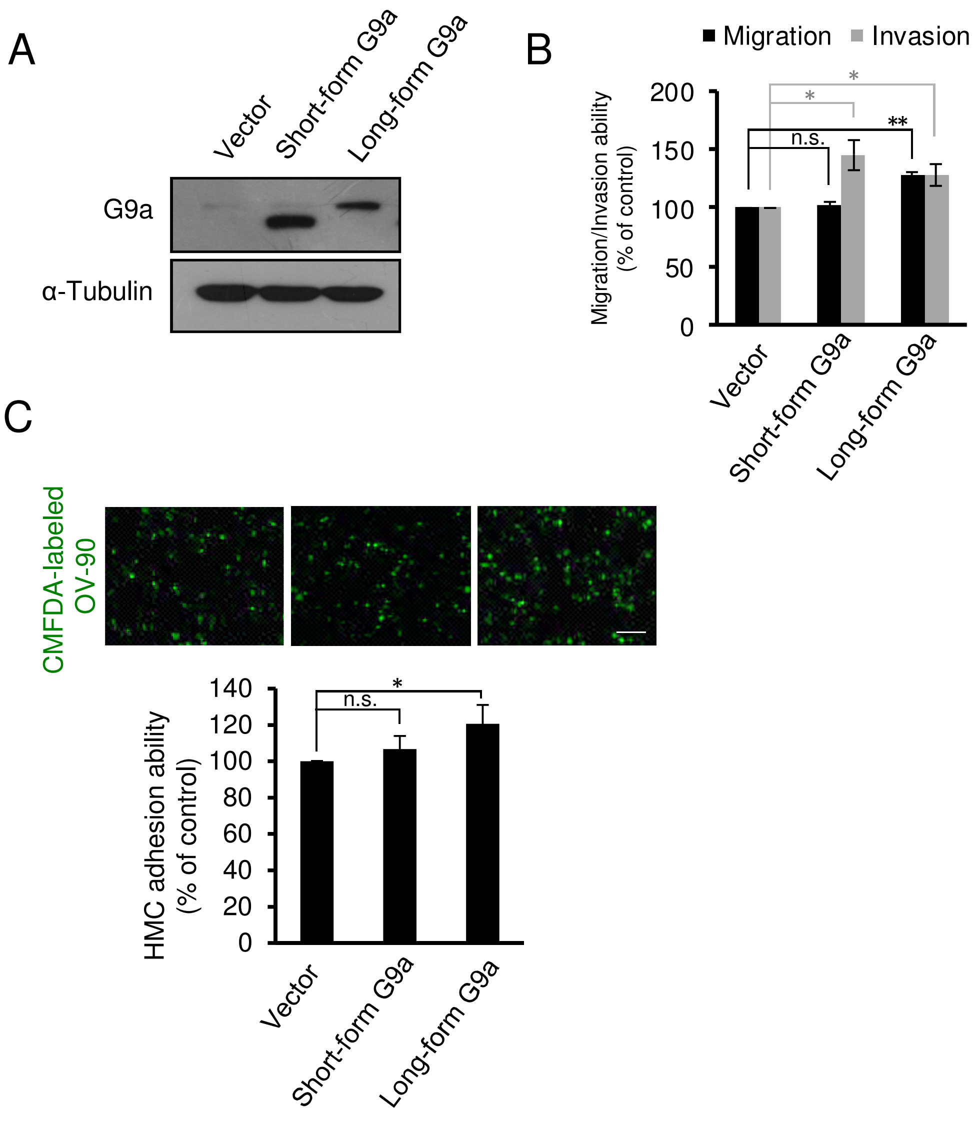

Figure S5. UNC0638 suppress invasion and induce G9a-suppressed genes expression in SKOV-3 cells.

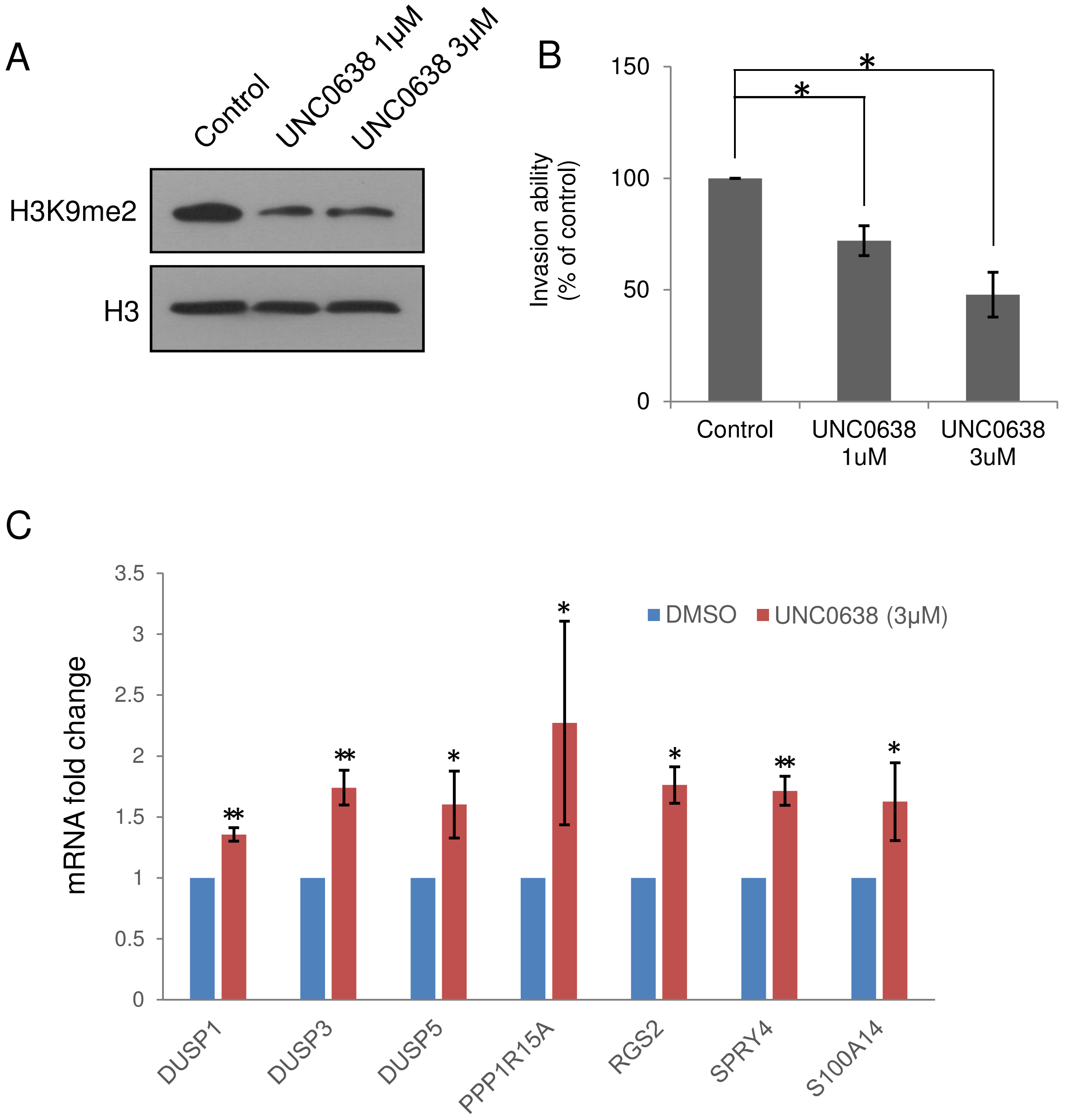

Figure S6. The occupancies of G9a on promoter regions of *SPRY4* and *DUSP5*.

A

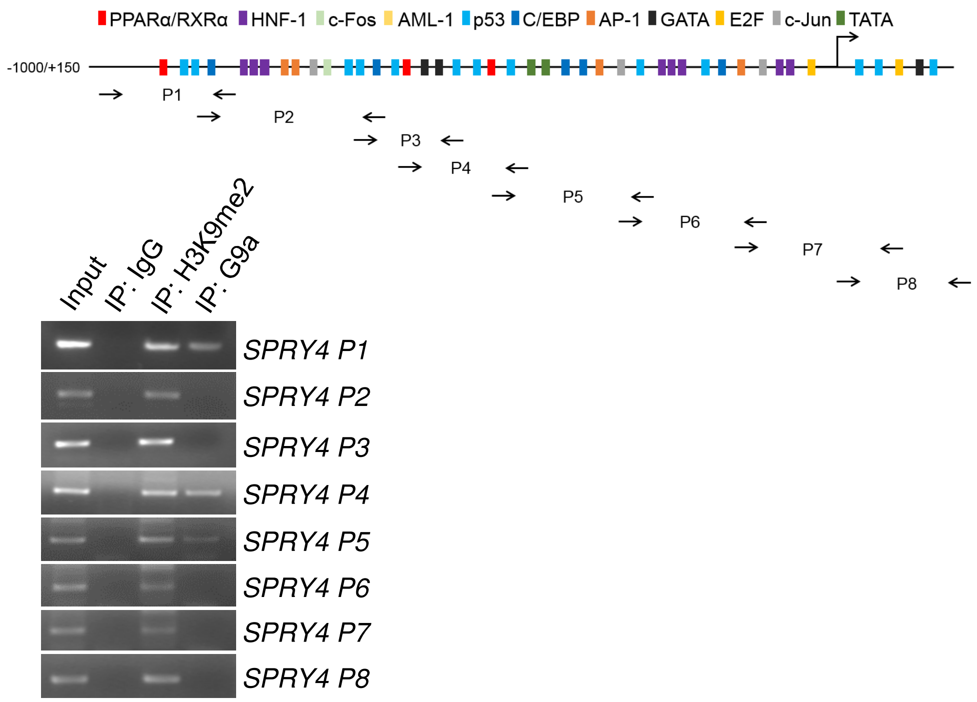

B

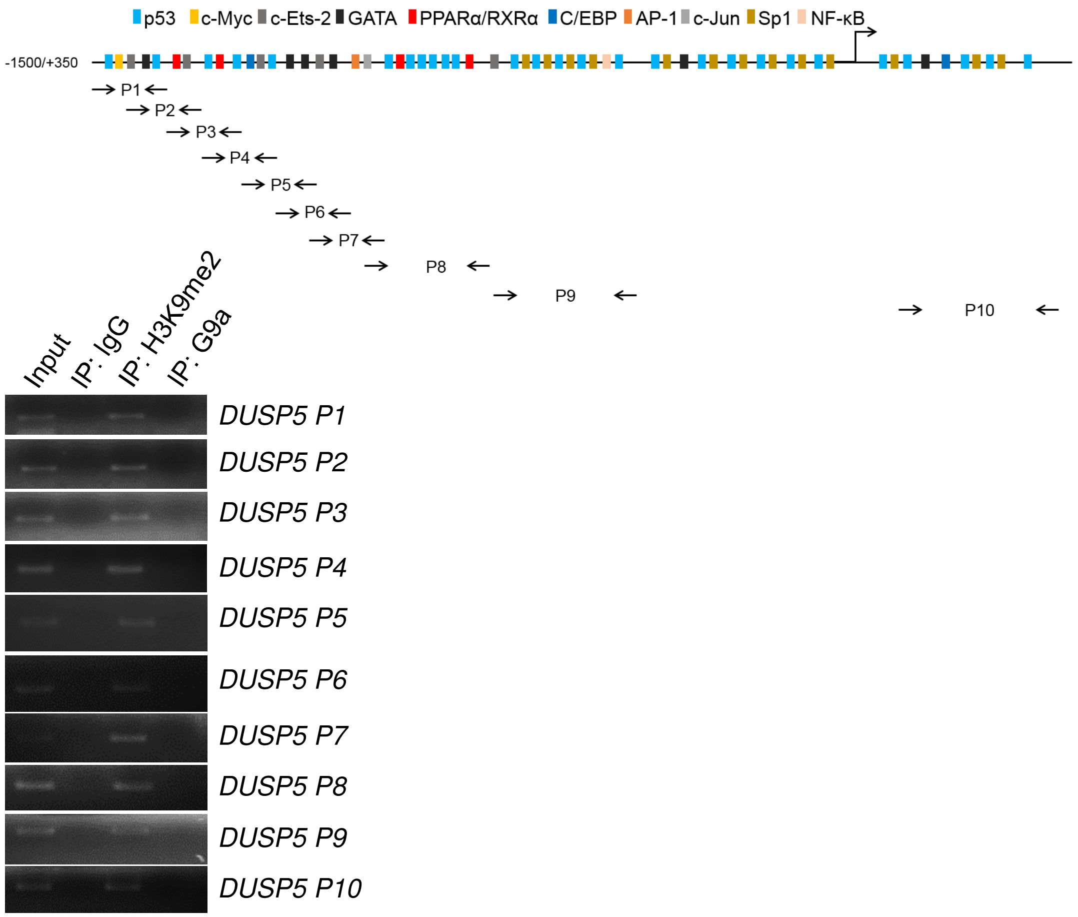

Figure S7. G9a and E-cadherin expressions inversely correlated with each other.

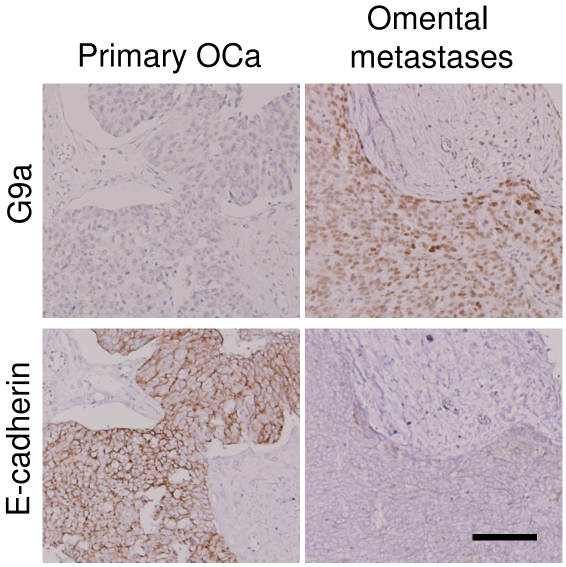

Figure S8. Knockdown of E-cadherin rescue G9a-depletion-induced cell invasion.

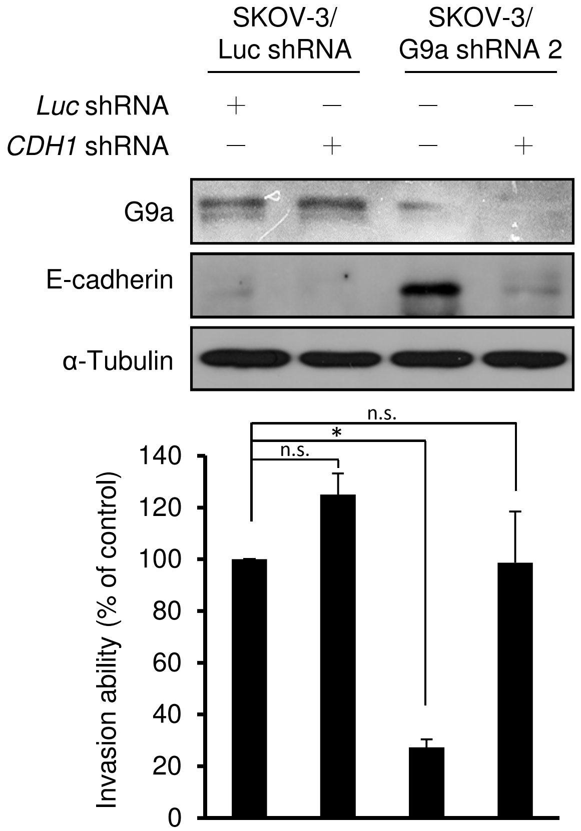

Supplement: Supplementary file 1 — Additional file 1: Figure S1-S8, Table S1-S6: Additional evidences support that G9a is a marker of aggressive ovarian cancer that promotes peritoneal metastasis. (PDF 720 KB) [file 12943_2014_1392_MOESM1_ESM.pdf]
